# Normal Haemostasis, Inherited Bleeding Disorders and Surgery: What Does the Anaesthesiologist Need to Know?

**DOI:** 10.3390/medicina61061087

**Published:** 2025-06-13

**Authors:** Mihai Ștefan, Daniela Filipescu, Cornelia Predoi, Liana Văleanu, Ștefan Andrei, Dana Tomescu

**Affiliations:** 1 Discipline of Anaesthesiology, “Carol Davila” University of Medicine and Pharmacy, 020021 Bucharest, Romania; 2 “Prof. C. C. Iliescu” Emergency Institute for Cardiovascular Diseases, 022328 Bucharest, Romania; 3 “Bichat Claude Bernard” University Hospital, 75018 Paris, France; 4 Fundeni Clinical Institute, 022328 Bucharest, Romania

**Keywords:** inherited bleeding disorders, haemophilia, haemostasis, von Willebrand disease, surgery

## Abstract

Haemostasis is a critical physiological process that ensures blood loss is minimised following vascular injury. Understanding the mechanisms of normal haemostasis is essential for managing patients with inherited bleeding disorders, particularly in the surgical setting. Inherited bleeding disorders, such as haemophilia and von Willebrand disease (vWD), pose unique challenges for anaesthesiologists and surgeons due to the increased risk of excessive bleeding during and after surgery. This state-of-the-art review outlines the essential knowledge for anaesthesiologists regarding normal haemostasis, the pathophysiology of inherited bleeding disorders, and the perioperative management strategies required for these patients. It draws on existing literature and current clinical guidelines to offer practical approaches for assessing and managing bleeding risks in surgical settings.

## 1. Introduction to Haemostasis

**Figure 1:**
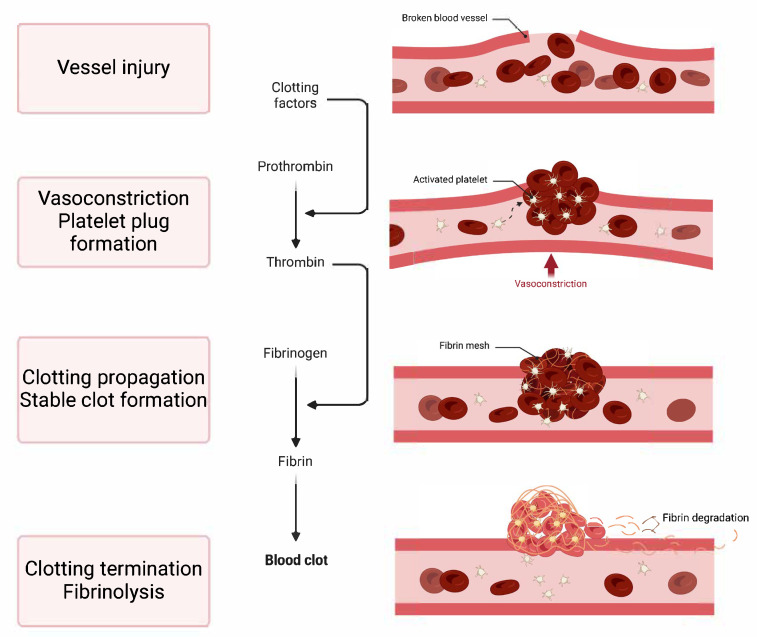
General view of haemostasis. Created in BioRender.com. Stefan, M. (2024) BioRender.com/u56q111 (accessed on 9 June 2025).

Haemostasis is the body’s intrinsic method for stopping blood flow after a vascular injury, ensuring that the circulatory system remains intact. This essential mechanism unfolds through a sequence of systematic steps that halt bleeding from a damaged blood vessel and facilitate its eventual repair. The process can be succinctly broken down into four primary actions [1,2] (Figure 1):

- Vasoconstriction: This is the immediate narrowing of the blood vessel, which reduces blood flow to the injured area.
- Platelet Plug Formation: At the injury site, platelets stick, become activated, and clump together to form a provisional plug that closes off small tears in the damaged vessel.
- Clotting Propagation: This phase involves strengthening the platelet plug with fibrin, which produces a stable clot. This step occurs through the coagulation cascade, a series of enzyme-driven reactions that end with the creation of fibrin, an insoluble protein.
- Clotting Termination and Fibrinolysis: The haemostatic process is intricately controlled by opposing mechanisms that inhibit excessive clotting (thrombosis) and guarantee the disintegration of clots as tissue repair progresses (fibrinolysis). Imbalances in haemostasis can lead to either haemorrhage or abnormal clot formation.

## 2. Formation of the Platelet Plug 

### 2.1. Platelet Adhesion

**Figure 2:**
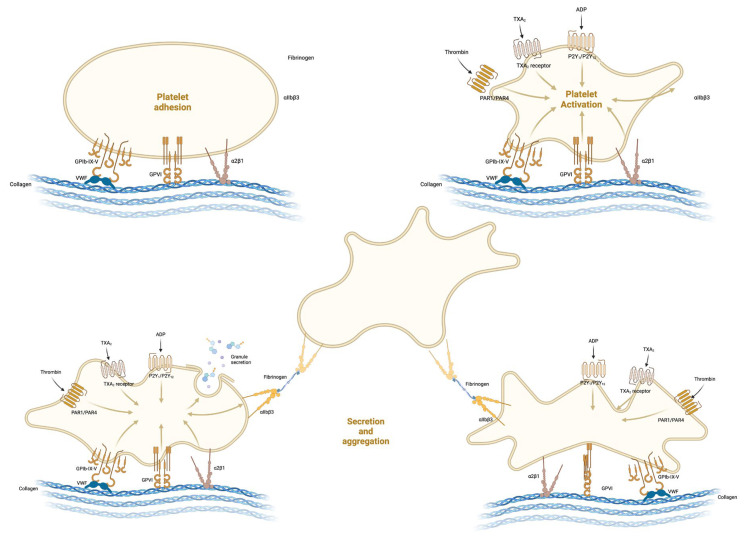
Formation of the platelet plug. Created in BioRender. Stefan, M. (2024) BioRender.com/d28c946 (accessed on 9 June 2025).

When the endothelial layer is compromised, it leads to the revelation of subendothelial collagen to which the von Willebrand factor (vWF) adheres [3]. Platelets in the bloodstream subsequently bind to the vWF through their GPIb receptors (part of the GPIb/IX/V complex) [3,4]. This binding process is essential as it triggers the haemostatic response when vascular damage occurs (Figure 2). The interaction becomes stronger in the presence of high shear stress, which is common in arterioles. Following the initial contact via GPIb, the platelets firmly anchor themselves to the collagen using GPVI and integrin α2β1, solidifying their connection to the damaged location [1].

### 2.2. Activation and Shape Change

Platelets that have adhered to an injury site become activated through the local liberation of agonists such as adenosine diphosphate (ADP), thromboxane A2, and serotonin, in addition to the influence of high shear stress [5]. This activation induces a transformation in their shape from discoid to stellate, enabling them to spread and more extensively cover the area of injury.

### 2.3. Platelet Secretion

The process of platelet secretion, a critical component of platelet activation during haemostasis, entails the release of granule substances into the plasma to enhance the haemostatic response, such as an array of proteins such as platelet factor 4, β-thromboglobulin, thrombospondin, fibronectin, vWF and platelet-derived growth factor [1,5]. These proteins are instrumental in vascular healing and in the further activation and summoning of platelets.

Platelets experience internal modifications upon activation, leading to the granules centralising and merging with the platelet plasma membrane, thereby discharging their contents.

The liberation of granule constituents has several significant consequences [1,3,5]:

- Recruitment: Compounds such as ADP and serotonin attract additional platelets to the injury site, encouraging their adherence and activation.
- Amplification: ADP’s release is particularly critical for the enhancement of platelet activation. It binds to P2Y1 and P2Y12 receptors on platelets, prompting a shape change and further granule release, thereby creating a positive feedback loop.
- Aggregation Enhancement: Thromboxane A2, synthesised by platelets, not only promotes platelet aggregation but also causes vasoconstriction, thereby diminishing blood flow to the injured area.
- Stabilisation: Proteins such as thrombospondin aid in solidifying the platelet mass by binding to fibrinogen and other extracellular matrix elements.
- Vessel Repair and Remodelling: Growth factors promote the healing and rebuilding of the vessel wall.

### 2.4. Platelet Aggregation

Platelets come together to form a clump through the cross-linking of fibrinogen, which binds to the activated glycoprotein IIb/IIIa receptors on adjacent platelets. The culmination of platelet adhesion and activation is the formation of a platelet plug at the injury site, a process coordinated by intricate interactions among GP receptors, soluble mediators, and adhesive proteins [1,3].

The pivotal moment in platelet aggregation is the activation of the GP IIb/IIIa receptor, or integrin αIIbβ3. This receptor is in a dormant state on inactive platelets. Activation is triggered by intracellular signals, including a rise in cytosolic calcium and the action of ADP and thromboxane A2, which induces a conformational shift in GP IIb/IIIa, heightening its affinity for fibrinogen and other adhesive proteins like vWF [1,2,3].

Fibrinogen acts as a critical connector in aggregation, binding to GP IIb/IIIa receptors on separate platelets, thus linking them. Its bivalency allows it to cross-link adjacent platelets, playing a vital role in the development of the platelet plug [1,2,3].

Calcium ions are indispensable for the conformational alteration in GP IIb/IIIa needed for fibrinogen attachment. The activation-induced surge in intracellular calcium is essential for the ‘inside-out’ signalling that activates GP IIb/IIIa.

Various soluble mediators intensify aggregation during the release reaction phase (mostly ADP and Thromboxane A2), and while initially the platelet plug is somewhat delicate, the activation of the coagulation sequence and thrombin generation turns fibrinogen into fibrin. Fibrin creates a network that solidifies the platelet plug into a sturdier clot [1,2,3,4].

Aggregation is meticulously controlled to avert excessive clotting, through prostaglandin I2 (Prostacyclin) and Nitric Oxide (NO), emitted by endothelial cells, work to inhibit platelet aggregation, and encourage vasodilation [1,2,3,4].

## 3. The Coagulation Cascade and Resolution of Clotting

**Figure 3:**
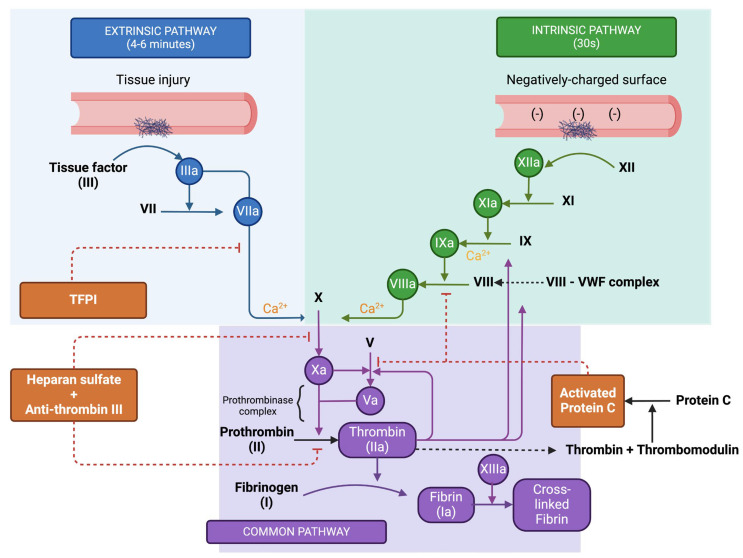
Coagulation cascade and regulation. Created in BioRender. Stefan, M. (2024) BioRender.com/b38c128 (accessed on 9 June 2025).

The coagulation cascade is a pivotal aspect of haemostasis, which solidifies the initial platelet plug and results in the formation of a permanent clot [2,6]. This cascade is a sequence of enzyme-driven reactions that transform soluble fibrinogen into insoluble fibrin strands (Figure 3). For anaesthesiologists, comprehending this cascade is vital, particularly in the management of coagulation disorders or when applying anticoagulant treatments. The coagulation cascade is typically categorised into three pathways: the intrinsic pathway, which is activated by internal trauma, the extrinsic pathway, which is triggered by external trauma, and the common pathway, where both intrinsic and extrinsic pathways converge to complete the clot formation process [4,6].

The intrinsic pathway begins when blood interacts with negatively charged surfaces, like the collagen found at an injury site or on artificial entities such as catheters or heart-lung machines. It incorporates the sequential activation of clotting factors XII, XI, IX, and VIII [2,4,6]:

- Factor XII, known as Hageman factor, is converted to its active form XIIa.
- This active form, Factor XIIa, then activates Factor XI to XIa.
- Factor XIa proceeds to activate Factor IX to IXa.
- Factor IXa, coupled with its cofactor Factor VIIIa (activated by thrombin), forms a complex that activates Factor X to Xa.

The extrinsic pathway is set in motion by external injuries that cause blood to leak from the vascular system and is associated with tissue factor (TF) or tissue thromboplastin [1,2,4,6]:

- Tissue factor, a protein found on subendothelial cells outside the blood vessels, associates with Factor VII, resulting in its activation to VIIa.
- The TF-VIIa complex then directly activates Factor X (Xa), bypassing the intrinsic pathway steps.
- The common pathway is the juncture at which the intrinsic and extrinsic pathways merge, culminating in the formation of a fibrin clot. This is facilitated by a large molecular complex known as prothrombinase. The steps in the common pathway include [2,4,6]:
- Factor Xa, paired with its cofactor Factor Va, assembles into the prothrombinase complex.
- Prothrombinase catalyses the transformation of prothrombin (Factor II) into thrombin (Factor IIa).
- Thrombin, in turn, acts on fibrinogen to convert it into fibrin.
- The resulting fibrin monomers then polymerize, forming a stable meshwork. This meshwork is further solidified by Factor XIIIa, which is activated by thrombin, and creates covalent linkages among the fibrin strands.

### 3.1. Regulation of the Coagulation Cascade

Endothelial cells are key to maintaining haemostatic equilibrium, ensuring that coagulation is balanced with anti-coagulation. They produce proteins that halt coagulation and prevent its spread beyond the injury site. The primary regulators of coagulation presented on the endothelial cell surface include [1,2]:

- Heparan sulphate (HS), which attaches to antithrombin (AT). This interaction induces a structural shift in AT, significantly enhancing its ability to bind with thrombin and Factor Xa, leading to their deactivation.
- Tissue factor pathway inhibitor (TFPI) targets the extrinsic tenase complex, neutralising it and inhibiting its activity.
- Thrombomodulin (TM) connects to thrombin, which is abundantly produced during the coagulation propagation phase. Binding to TM alters thrombin’s structure, which in turn activates protein C (aPC). Once activated, aPC, with protein S as a supporting factor, attaches to Factor Va, rendering it inactive.

### 3.2. Cell Based Model of Coagulation

While the classical coagulation cascade mirrors the process observed in laboratory tests, it does not fully represent the mechanism of haemostasis in living systems. The cell-based model of coagulation, which places thrombin at the core, offers a more accurate depiction of in vivo coagulation [3]. Thrombin not only plays a role in its own generation and regulation through feedback mechanisms but also in the transformation of fibrinogen into a cross-linked fibrin network [1,3].

In the cell-based model, certain proteins traditionally associated with the intrinsic pathway, like factor XII and prekallikrein, are recognised to have a minimal contribution to coagulation in vivo and are therefore excluded [1,2,3]:

- During the initiation phase, coagulation is instigated by vessel damage that exposes tissue factor (TF) in the plasma. This phase unfolds on a TF-bearing cell, leading to the activation of factors V and VII in proximity, which subsequently activate other clotting factors, resulting in the generation of a modest quantity of thrombin. Moreover, platelets passing by become activated by both TF and vWF, as well as by the initial thrombin generated.
- The amplification phase sees the further activation of clotting factors and platelets in readiness for the extensive production of thrombin. Thrombin binds to the GP1b receptors on the platelet surface, activating factors XI, VIII, and V.
- The propagation phase takes place on the surface of an activated platelet, which is primed with activated clotting factors. This platelet acts as a catalyst, leading to the production of substantial amounts of thrombin.

The thrombin produced then converts fibrinogen (factor I) into fibrin (factor Ia), which, through the GPIIb/IIIa receptor, cross-links platelets, contributing to the stability of the resulting clot [1].

### 3.3. Resolution of Clotting

In the physiological process of fibrinolysis, fibrin within blood clots undergoes gradual dissolution. This mechanism is integral to wound healing and is essential for maintaining the patency of small blood vessels, and is dependent on plasminogen, a proenzyme which is incorporated into the developing fibrin matrix, subsequently turning to plasmin, which acts on fibrin, generating fibrin degradation products (FDPs), including D-dimer. Clinically, D-dimer levels are frequently measured to assist in the diagnosis of venous thromboembolic disorders, such as pulmonary embolism [1,2,4].

## 4. Inherited Bleeding Disorders

### 4.1. Disorders of Primary Haemostasis

**Von Willebrand Disease (vWD)**

Von Willebrand Disease is the most prevalent inherited bleeding disorder (reported incidence 0.6–1.3%), characterised by a deficiency or dysfunction of vWF, a key protein in platelet adhesion [5]. vWD is classified into three main types, with varying degrees of severity [5,6]:

- Type 1 vWD, the most common and mildest form, typically presents with mucocutaneous bleeding, such as epistaxis, menorrhagia, and bleeding after dental extractions.
- Type 2 vWD involves qualitative defects in vWF and presents with a similar but often more severe bleeding phenotype, and has four subtypes (2A, 2B, 2N, 2M).
- Type 3 vWD, the most severe form, results from a near-total lack of vWF and presents with significant bleeding risks.

**Platelet Function Disorders**

Inherited disorders of platelet function encompass a range of conditions that affect platelet adhesion, activation, and aggregation, despite normal platelet counts. Clinically important are the following [5,7,8]:

- Glanzmann Thrombasthenia: A rare condition characterised by the absence or dysfunction of the GPIIb/IIIa receptor, crucial for platelet aggregation. Patients typically present with mucocutaneous bleeding, such as gum bleeding, epistaxis, and menorrhagia, and may experience severe bleeding after surgical procedures.
- Bernard-Soulier Syndrome: Another rare disorder, marked by a deficiency of the GPIb-IX-V complex, essential for platelet adhesion to vWF. This condition presents similarly to Glanzmann Thrombasthenia but can be distinguished by the presence of giant platelets on a blood smear.
- Platelet type Von Willebrand Disease (also known as pseudo vWD) is a distinct entity, characterised by an abnormal interaction between platelets and vWF. This disorder is caused by a gain-of-function mutation in the platelet glycoprotein Ibα, leading to enhanced binding to vWF. Patients present with a clinical phenotype similar to Type 2B vWD, including thrombocytopenia and mucocutaneous bleeding. Diagnosis can be challenging and requires specific laboratory assays to differentiate it from Type 2B vWD, given the similar clinical and laboratory profiles.
- Storage pool disorders (SPDs) are a group of inherited platelet function disorders characterised by deficiencies in platelet granules or their contents. These disorders affect the storage and release of critical haemostatic agents from platelets, leading to bleeding symptoms.

### 4.2. Disorders of Secondary Haemostasis

**Haemophilia**

Haemophilia A (factor VIII deficiency) and Haemophilia B (factor IX deficiency) are the most prevalent inherited coagulation disorders, affecting primarily males due to their X-linked recessive inheritance pattern [5,6]. The severity of the disease is inversely proportional to the activity levels of the respective clotting factors and is categorised as mild, moderate, or severe (Table 1) [9].

Patients with haemophilia may present with spontaneous bleeding into muscles and joints (hemarthrosis), easy bruising, and prolonged bleeding after trauma or surgical procedures [9,10]. Severe forms can lead to chronic joint disease and potentially life-threatening haemorrhages.

Von Willebrand Disease (vWD) is often discussed in the context of primary haemostasis due to its role in platelet function; however, it also plays a significant role in secondary haemostasis through its carrier function for factor VIII. Deficiency or dysfunction of vWF can lead to reduced factor VIII levels, contributing to a secondary haemostasis disorder [12].

**Rare Bleeding Disorders**

Other rare inherited disorders of secondary haemostasis include deficiencies of factors I (fibrinogen), II (prothrombin), V, VII, X, and XIII, as well as combined FV+FVIII deficiencies. These conditions vary in their clinical presentation from mild to severe bleeding tendencies [13,14]. Isolated factor XI deficiency, also known as Haemophilia C, is an autosomal recessive disorder that can affect both males and females, with variable bleeding tendencies [15]. It is more common in certain populations, such as Ashkenazi Jews. The bleeding risk in Factor XI deficiency is less predictable than in Haemophilia A or B, with some patients experiencing significant bleeding following surgical procedures, while others may have minimal or no bleeding issues.

## 5. Perioperative Anaesthetic Considerations

### 5.1. Preoperative Evaluation—Bleeding Assessment Tools

Individuals with genetic bleeding conditions face an increased risk of bleeding during surgery, even when the condition is considered mild [16]. Preoperative identification of these disorders is possible with the application of a Bleeding Assessment Tool (BAT), which involves a systematic patient interview and a scoring system that evaluates the intensity of each reported bleeding symptom to calculate a cumulative bleeding score (BS) [17]. Nevertheless, to date, there has been no preoperative bleeding diathesis questionnaire that has been generally accepted for clinical use.

The World Federation of Haemophilia’s compendium of assessment tools [18] gives as alternatives for BATs various scores, such as the Molecular and Clinical Markers for the Diagnosis and Management of Type 1 (MCMDM-1) vWD Bleeding Questionnaire [19], the Paediatric Bleeding Questionnaire (PBQ), which is dedicated to children with vWD [20], the Šrámek Bleeding Score [21] and the International Society on Thrombosis and Haemostasis/Scientific and Standardization Committee Bleeding Assessment Tool (ISTH BAT) [22]. Of these, the ISTH BAT has been most frequently researched in the perioperative context.

There is ample evidence that the ISTH BAT is well suited for detecting patients with vWD [16], IPDs [23,24,25], haemophilia A and B [26] and even inherited F VII deficiency [27].

The ISTH BAT was, not, however, able to predict the risk of future bleeding in patients with suspected IBDs [28], nor is it, based on current evidence, able to discriminate in the context of elective surgery between patients with and without haemostatic abnormalities [29,30].

In a study evaluating the implementation of Patient Blood Management in cardiac surgery, using a preoperative standardised haemostasis questionnaire was an independent negative predictor for blood transfusion perioperatively. However, this is not validated in the context of IBDs [31].

Current European Society of Anaesthesiology and Intensive Care (ESAIC) guidelines suggest the use of BATs for detecting and predicting the risk of bleeding in patients in whom IBDs are suspected and who are scheduled for surgery, but the class of recommendation and level of evidence are relatively low (2B) [17].

### 5.2. Preoperative Evaluation—Laboratory Tests

An outline of in vitro diagnostic options is available in Table 2.

When there is a suspicion of von Willebrand disease (vWD), the standard laboratory screenings include tests for vWF antigen, activity of ristocetin cofactor, as well as the activity of Factor VIII coagulant [32]. However, a diagnosis of vWD cannot be solely based on a low vWF level, as such levels can also be present in individuals without the disease, particularly among those with type O blood [33].

The diagnosis of IPDs can be complex and may require referral to a haematologist, particularly one who specialises in bleeding disorders [7,8]. It is important to note that not all platelet function disorders will be detected by standard tests, and in some cases, diagnosis can be challenging. Laboratory investigations include a complete blood count and a peripheral blood smear to examine platelet count and morphology [34]. Functional assessments are conducted using platelet aggregation and secretion tests, flow cytometry for surface protein analysis, and the PFA-100 test to measure platelet plug formation [35]. Genetic testing is utilised to confirm specific disorders by detecting mutations in the relevant genes.

Haemophilia A and B are diagnosed through initial screening tests and specific factor assays. The process begins with a clinical assessment, focusing on symptoms like spontaneous or prolonged bleeding and family history. Initial blood tests include the activated Partial Thromboplastin Time (aPTT), which is typically prolonged in haemophilia, while the Prothrombin Time (PT) remains normal [9,10]. The intrinsic clotting factors are further evaluated to determine the specific type of haemophilia; Factor VIII assay is used for Haemophilia A, and Factor IX assay for Haemophilia B, with the severity of the condition defined by the activity level of these factors.

Further laboratory tests may include inhibitor screening to detect antibodies against the deficient factor, which are crucial for planning treatment [36]. Genetic testing can also be conducted for precise mutation identification, useful for family planning and understanding inheritance patterns. Mixing studies may be conducted to discern between a factor deficiency and the presence of an inhibitor. A haematologist typically oversees the diagnostic process, which not only confirms the diagnosis but also informs the treatment approach.

### 5.3. Management Strategies

Individuals with genetic bleeding conditions have an elevated risk of bleeding during and after surgery [8,37]. It is crucial for their treatment to involve a haematologist and to be conducted in specialised facilities that are well-versed in coagulation disorders. According to the latest guidelines from the World Federation of Haemophilia (WFH), as well as the European guidelines on severe bleeding management, surgeries for patients with haemophilia should be conducted with the support of a comprehensive haemophilia treatment centre or in close consultation with one [10,17].

Additionally, despite their predisposition to bleeding, patients with inherited bleeding disorders (IBDs) may also encounter thromboembolic events post-surgery. Evidence from a substantial surgical database indicated that while patients with von Willebrand disease (VWD) have a higher incidence of postoperative bleeding compared to those without VWD, they experience major cardiac and thrombotic complications during the perioperative period at similar rates [37]. To this end, postoperative thromboprophylaxis should not be overlooked in these patients, providing factor levels are kept within therapeutic range. WFH guidelines recommend the balancing of bleeding and thrombotic risk for each individual patients, and using primarily mechanical thromboprophylaxis, while reserving or pharmacological thromboprophylaxis for high-risk patients, after haemostatic control is deemed appropriate [10].

### 5.4. Regional Anaesthesia

Current recommendations state that neuraxial anaesthesia should be avoided in haemophilic patients with factor levels below 50 IU/dL (corresponding to 50% factor activity level). There is, however, little data to guide practice for peripheral nerve blocks, but general good clinical practice should prevail (e.g., use ultrasound guidance for all patients, monitor factor levels before procedure).

### 5.5. Haemostatic Interventions—Substitutions

It is accepted that preoperatively, haemostatic corrections should be tailored to particular disorders but also to the type of surgery and its inherent bleeding risk, as well as other individual factors, such as bleeding phenotype (Table 3).

A comprehensive review designed to inform clinical guidelines for von Willebrand disease (VWD), suggests that maintaining factor VIII (FVIII) and vWF levels above 0.50 IU/mL for a minimum of three days post-surgery can lead to effective haemostasis, which was deemed excellent by the researchers in 74–100% of the surgeries analysed [38]. However, there is a significant degree of uncertainty associated with this evidence, indicating that further research might be needed to confirm these findings. Accordingly, the vWD guidelines suggest tailoring the duration and specific target levels of therapeutic interventions to the individual, factoring in the patient’s history, the nature of the procedure, and the patient’s bleeding history, as well as the accessibility of vWF and FVIII assays [39].

There is a notable lack of high-quality research concerning patients with haemophilia, leading to an absence of agreement regarding the best replacement therapy and the exact haemostatic levels needed for each factor. Some data have indicated that there is minimal difference in surgical haemostasis and patient outcomes when comparing low-dose therapy to the standard protocols previously suggested by the WFH [40]. Nonetheless, the latest WFH guidelines have shifted towards recommending tailored pharmacokinetic monitoring to fine-tune the treatment approach for individuals [10].

Depending on the type of surgery (minor vs. major) and practice pattern (higher-dose or lower-dose), factor replacement should be adjusted depending on pre-procedural patient level. For haemophilia A, WFH guidelines suggest that higher-dose treatment approaches aim for initial peak FVIII levels of 80–100 IU/dL for major surgeries and 50–80 IU/dL for minor surgeries [10]. Lower-dose approaches target initial peak FVIII levels of 60–80 IU/dL for major surgeries and 40–80 IU/dL for minor surgeries. In haemophilia B, higher-dose strategies seek peak FIX levels of 60–80 IU/dL initially for major surgeries and 50–70 IU/dL for minor surgeries [10]. Lower-dose strategies aim for initial peak FIX levels of 50–80 IU/dL for major surgeries and 40–80 IU/dL for minor surgeries [41].

A recent paper on cardiac surgery patients suggests that perioperative targets in haemophiliacs should be titrated to WFH higher levels, with prolongation of monitoring and factor replacement for up to 14 days postoperatively (Table 4) [42], a duration which is confirmed by other authors as well [43].

For individuals with HA with high-reactive inhibitors to FVIII undergoing surgical or other invasive interventions, the WFH suggests the use of bypassing agents (BPAs), such as recombinant activated factor VII (rFVIIa) or activated prothrombin complex concentrate (aPCC), depending on the treating physician’s judgement [36]. In cases of haemophilia B (HB) with factor IX (FIX) inhibitors requiring surgery, the WFH prefers rFVIIa over aPCC due to the presence of FIX in aPCC, which can potentially trigger or exacerbate an allergic response [10]. Nonetheless, evidence is insufficient to definitively conclude which agent is superior or to establish the best dosing strategy.

While in previous guidelines from the ESAIC fresh frozen plasma and cryoprecipitate were seen as viable options for patients with vWD in the absence of factor concentrates, the 2023 edition does not recommend this alternative for treatment. In the case of haemophiliacs, given their low concentrations of coagulation factors, there is no evidence of any benefit when treating patients with either FFP or cryoprecipitate [17,44,45].

Prophylactic platelet transfusion in patients with IPDs is not routinely recommended, as evidence in the literature is conflicting. The latest European guidelines on severe bleeding management did not make a clear recommendation in these cases, and the prudent approach seems to either direct these patients to referral centres or work in close contact with one for the adequate planning of surgical procedures [17].

### 5.6. Haemostatic Interventions—Desmopressin

Desmopressin (DDAVP) serves as a therapeutic agent in type 1 von Willebrand disease (vWD) by promoting the endogenous release of vWF from endothelial cell stores, thereby transiently augmenting plasma vWF and factor VIII levels [46]. The elevation in vWF not only enhances factor VIII stability but also optimises platelet function through improved adhesion and aggregation, crucial for effective haemostasis [47]. The administration of DDAVP, which can be given intravenously, subcutaneously, or intranasally, is tailored to the patient’s demonstrated response, with efficacy being time-limited and most pronounced in the hours following administration [48]. It is noteworthy that DDAVP’s effectiveness is predominantly in type 1 vWD, with limited to no efficacy in type 3 and certain subtypes of type 2 vWD, where the vWF protein is either absent or dysfunctional, respectively [39].

DDAVP, which elevates plasma concentrations of vWF and factor VIII, may be preferred for managing mild HA, provided it can elevate FVIII to satisfactory therapeutic thresholds [49]. The WFH advocates for its use in achieving perioperative haemostasis in patients who exhibit a positive response during preoperative assessment [10]. It is essential to evaluate the drug’s efficacy in each individual prior to surgery due to the considerable interpatient variability in response.

DDAVP standard dose is 0.3 mcg/kg of body weight, with a maximal dose capped at 30 mcg, above which no supplementary activity of FVIII is obtained [50,51].

In patients with IBDs other than VWD or HA, the precise quantification of the response to DDAVP is not well-defined, and its use is thus based on empirical observation. The latest guidelines from the ESAIC do not recommend DDAVP for such patients, given the lack of data [17].

### 5.7. Haemostatic Interventions—Antifibrinolytics

Antifibrinolytics (AFs), such as tranexamic acid and aminocaproic acid, are medications that can play a role in the management of IBDs blocking the activation of plasminogen to plasmin, a key enzyme responsible for clot breakdown [52]. Through this mechanism, antifibrinolytics help to stabilise the formation of the fibrin clot, thus preventing or reducing excessive bleeding.

The latest ESAIC guidelines recommend the use of AFs in the perioperative setting for patients with haemophilia or vWD, as adjuncts to factor replacement, as they have been proven in several trials to reduce bleeding when compared to factor replacement alone [17,53,54].

AFs can also be used alone in such patients when undergoing minor mucosal or dental procedures, and this recommendation is also endorsed by the WFH, but based on a low quality of evidence [39,54].

As for DDAVP, the use of AFs in IPD is based on experience, rather than evidence [55].

### 5.8. Haemostatic Interventions—RFVIIa

RFVIIa serves as an essential therapeutic option for managing bleeding in haemophilia patients with inhibitors to factors VIII or IX, by initiating a factor VIII/IX-independent mechanism of haemostasis [36,56,57]. It achieves this by directly activating factor X in the presence of activated platelets, facilitating the thrombin-mediated conversion of fibrinogen to fibrin, thus contributing to the formation of a stable haemostatic plug at the site of vascular injury [58]. RFVIIa’s haemostatic efficacy contingent upon adequate platelet function and fibrinogen levels [56].

Its off-label use in other disorders than haemophilia with inhibitors has been described and the ESAIC guidelines suggest it can be a viable option in patients with Glanzmann thrombasthenia, based on registry data, as well as the SPATA study [8,17,59,60].

An outline of the history of the recommendations from ESAIC 2013, 2017 and 2023 guidelines on severe bleeding management is available in Table 5.

### 5.9. Emergency Surgery in Patients with Inherited Bleeding Disorders

The perioperative management of patients with inherited bleeding disorders (IBDs) undergoing emergency surgery poses significant challenges due to the limited time available for diagnostic confirmation and haemostatic optimisation. In such cases, clinical history (including any known diagnosis, bleeding phenotype, or previous haemostatic treatment) becomes paramount, and a prompt multidisciplinary approach involving anaesthesiologists, haematologists, and surgeons is essential. For known haemophilia patients, immediate administration of factor VIII or IX concentrates (or bypassing agents in case of inhibitors) is warranted to achieve target levels before incision, as described above. In suspected von Willebrand disease or platelet disorders, antifibrinolytics (e.g., tranexamic acid) and desmopressin (in known responders) may be used as temporising measures, while awaiting laboratory confirmation. Given the increased bleeding risk, neuraxial techniques are generally contraindicated unless haemostatic status can be rapidly corrected. Postoperative monitoring and factor replacement should continue for a duration aligned with the surgical bleeding risk, and thromboprophylaxis must be individually tailored based on the adequacy of haemostasis. When available, emergency procedures should be performed in or in consultation with specialised haemophilia treatment centres to minimise complications and optimise outcomes.

## 6. Conclusions

The contemporary understanding of coagulation has shifted from the traditional in vitro classification of primary and secondary haemostasis to a more nuanced cell-based model that reflects the in vivo reality. This model emphasises four critical phases: initiation, amplification, propagation, and termination, each of which is essential for the proper function of haemostasis physiology. Furthermore, fibrinolysis is recognised as a natural and necessary process that serves to balance coagulation, preventing the formation of excessive clots. Anaesthesiologists must have a firm grasp of these mechanisms to fully comprehend the pathophysiology of inherited coagulation disorders and to effectively utilise pharmacological agents to manipulate haemostatic processes. This understanding is pivotal not only for the management of inherited bleeding disorders but also for the strategic control of coagulation during surgical procedures, ensuring patient safety and optimal clinical outcomes.

## Figures and Tables

**Table 1:** Classification of haemophilia by severity [10,11].

Severity of Disease	Factor Activity	Clinically Relevant Bleeds
Mild	5–40%	Major trauma, surgery, rarely spontaneous
Moderate	1–5%	Minor trauma, surgery, sometimes spontaneous
Severe	<1%	Spontaneous

**Table 2:** Available screening and diagnosis tests for IBDs.

Disease	Screening Test	Specific Tests
vWD	None	vWF antigen Ristocetin cofactor F VIII
IPD	None	CBC Peripheral blood smear Functional (aggregation and secretion) tests PFA-100
Haemophilia A	aPTT elevated PT normal	F VIII Inhibitor screening Genetic testing
Haemophilia B	aPTT elevated PT normal	F IX Inhibitor screening Genetic testing

Abbreviations: IBD—inherited bleeding disorder, vWD—von Willebrand disease, F VIII—coagulation factor VIII, IPD—inherited platelet disorders, CBC—complete blood count, PFA—platelet function assay, aPTT—activated partial thromboplastin time, PT—prothrombin time, F IX—coagulation factor IX.

**Table 3:** Specific and adjuvant therapies for IBDs.

Disease	Specific Treatment	Non-Specific, Adjuvant Treatment
vWD	vWF FVIII DDAVP in type 1	Antifibrinolytics
IPD	Platelet transfusion, not prophylactically	DDAVP Antifibrinolytics rVIIa in Glanzmann thrombasthenia
Haemophilia A	FVIII BPAs if high-reactive inhibitors rFVIIa or aPCC	DDAVP in mild HA Antifibrinolytics
Haemophilia B	FIX BPAs if high-reactive inhibitors rFVIIa	Antifibrinolytics

Abbreviations: vWD—von Willebrand disease, F VIII—coagulation factor VIII, DDAVP—desmopressin, IPD—inherited platelet disorders, rVIIa—recombinant factor VII, BPA—by-passing agent, aPCC—activated prothrombin complex concentrate, FIX—coagulation factor IX.

**Table 4:** Perioperative factor level targets in major surgery [10,41,42].

Haemophilia Type	Preoperative Level	Intraoperative Level	Days 1–3	Days 4–6	Days 7–14
Haemophilia A	80–100%	80–100%	60–80%	40–60%	30–50%
Haemophilia B	60–80%	60–80%	30–50%	30–50%	20–40%

**Table 5:** An evolution of ESAIC guideline recommendations, from 2013 to 2023, refs. [18,42,43].

Recommendations	2013	2017	2023
Referral to haematologist for assessment, planning and management	2C for preoperative assessment and planning 1C for vWD, IPDs and haemophilia perioperative management	2C for assessment 1C for perioperative management, in centres with expertise in IBDs	1B for perioperative management, in centres with expertise in IBDs
The use of bleeding assessment tools for detecting and predicting the perioperative risk of bleeding before surgery and invasive procedures in patients with suspected or confirmed IBDs.	1C	1C	2B
Individualised preoperative haemostatic correction depending on the specific disorder, type of surgery and individual factors (bleeding phenotype).			2C
Replacement/substitution therapy with factor concentrates, either plasma-derived or recombinant products, for major bleeding/surgery in patients with vWD or haemophilia A and B.	1C	1C	1C
For haemophilia patients with inhibitors—administer either rFVIIa or aPCCs.	2C	2C	2C
Routine perioperative platelet transfusion in patients with IPDs.	Against—2C	Against—2C	Against—2C
There is insufficient data to recommend routine perioperative supplementation of deficient factors in patients with rare bleeding disorders.	No recommendation	No recommendation	No recommendation
Desmopressin as a first-line treatment for minor bleeding/surgery, after a trial testing and in the absence of contraindications.	1C for vWD 2C for IPDs	1C for vWD 2C for IPDs and haemophilia A	2C for vWD and haemophilia A No recommendation for IPDs—insufficient data
Perioperative antifibrinolytics as adjunct therapy in haemophilia, vWD or IPDs.	2C	2C	2B for haemophilia, vWD.
Perioperative antifibrinolytics as monotherapy	-	-	2C—in patients with IPDs and in patients with haemophilia or vWD for minor mucosal or dental procedures
Recombinant factor VII activated considered in patients with Glanzmann thrombasthenia undergoing surgery	1C	1C	2C
Recombinant factor VII activated used in perioperative bleeding due to inherited factor VII deficiency.	2C	2C	2C
There is insufficient data to recommend the use of recombinant factor VII activated in perioperative bleeding for patients with other RBDs.	C	No recommendation	No recommendation
